# Insights into the Roles of Cyclophilin A During Influenza Virus Infection

**DOI:** 10.3390/v5010182

**Published:** 2013-01-15

**Authors:** Xiaoling Liu, Zhendong Zhao, Wenjun Liu

**Affiliations:** Center for Molecular Virology, Key Laboratory of Pathogenic Microbiology and Immunology, Institute of Microbiology, Chinese Academy of Sciences, Beijing 100101, China; E-Mails: xiaolingliu03@163.com (X.L.), mrzzdnew@163.com (Z.Z.)

**Keywords:** influenza virus, Cyclophilin A, Cyclosporin A, virus-host interaction

## Abstract

Cyclophilin A (CypA) is the main member of the immunophilin superfamily that has peptidyl-prolyl *cis-trans* isomerase activity. CypA participates in protein folding, cell signaling, inflammation and tumorigenesis. Further, CypA plays critical roles in the replication of several viruses. Upon influenza virus infection, CypA inhibits viral replication by interacting with the M1 protein. In addition, CypA is incorporated into the influenza virus virions. Finally, Cyclosporin A (CsA), the main inhibitor of CypA, inhibits influenza virus replication through CypA-dependent and -independent pathways. This review briefly summarizes recent advances in understanding the roles of CypA during influenza virus infection.

## 1. Introduction

Influenza virus is an enveloped negative-sense RNA virus that causes major public health problems worldwide. There are eight RNA segments in the inﬂuenza A virus encoding 14 viral proteins: the polymerase proteins (PB1, PB2, PA) [1,2], nucleocapsid protein (NP), hemagglutinin (HA), neuraminidase (NA), matrix proteins (M1 and M2), nonstructural proteins (NS1 and NS2) and the recently described PB1-F2, PB1 N40, PA-X and M42 proteins [1,2,3,4]. The M1 protein is the most abundant protein in the viral particle and forms the bridge between the viral envelope and core. M1 is a multifunctional protein in the influenza virus lifecycle that is involved in uncoating, transcription, the nuclear export of viral ribonucleoprotein complexes (vRNP), assembly and budding. Several host cell factors are putatively required for regulation of influenza virus replication via interaction with M1 at different stages of infection [5,6,7,8]. For instance, the peptidyl-prolyl *cis-trans* isomerase Cyclophilin A (CypA) participates in influenza virus infection at several steps [9,10,11,12]. In addition, as a ubiquitously expressed host factor, CypA plays important roles in several viral infections [9,11,13,14,15,16,17,18,19]. The aim of this review is to promote our understanding of the effects of CypA during influenza virus infection.

## 2. The Localization and Function of CypA

CypA is a ubiquitously distributed intracellular protein found in all tissues in mammals, and it possesses peptidyl-prolyl *cis-trans* isomerase activity [20]. CypA has chaperon-like activity and takes part in protein-folding processes [21]. Further, CypA is the major intracellular receptor for the immunosuppressive drug cyclosporin A (CsA), and the CsA-CypA complex interacts with and inhibits calcineurin, a calcium-calmodulin-activated serine/threonine-specific protein phosphatase [22], which in turn suppresses T-cell activation. In addition to its intracellular functions, CypA can be secreted into the extracellular environment [23,24]. Extracellular CypA acts as a growth factor in several cell types. Furthermore, CypA is also involved in the pathogenesis of several diseases, including viral infection, cardiovascular disease and cancer. The extracellular functions of CypA are mediated by CD147, which is expressed on a wide variety of cells, including hematopoietic, epithelial and endothelial cells [25,26,27,28,29]. CD147 is a widely expressed type I integral membrane protein that is implicated in many physiological and pathological activities. Drugs targeting either CD147 or Cyps demonstrate a significant anti-inflammatory effect in animal models of acute or chronic lung diseases, suggesting a therapeutic pathway for some diseases.

## 3. CypA as an Important Host Factor in Viral Infections

Accumulating evidence indicates that CypA is an important host factor for successful viral infection. CypA is involved in the lifecycle of several viruses, such as human immunodeficiency virus type 1 (HIV-1), influenza virus, hepatitis C virus (HCV), hepatitis B virus (HBV), vesicular stomatitis virus (VSV), vaccinia virus (VV), severe acute respiratory syndrome coronavirus (SARS-CoV) and Rotavirus (RV) [10,11,12,13,14,15,16,17,18,19,30,31]. CypA is also incorporated into several enveloped virus particles, such as HIV-1, influenza virus, VV and VSV [9,14,15,32,33]. However, the function of CypA in virus particles is still unclear.

CypA has been extensively studied from the gene to protein level during HIV-1 infection. CypA is encoded by the peptidyl prolyl isomerase A (*PPIA*) gene, and regulatory *PPIA* polymorphisms are a component of genetic susceptibility to HIV-1 infection or disease progression [34]. In addition, the interaction of CypA and capsid protein (CA) in newly infected human target cells usually aids viral infectivity [16,35]. Furthermore, the interaction of newly synthesized HIV-1 CA with CypA is required for HIV-1 to induce dendritic cell (DC) maturation [36]. CypA may interact with other HIV-1 proteins, such as Vpr and p6, to regulate HIV infection [37,38,39,40,41]. Finally, CD147 is the main receptor for CypA on human leukocytes [42], and the interaction of CD147 and CypA may regulate an early step of HIV-1 infection [43]. The interaction of CD147 with CypA induces MA phosphorylation to regulate the liberation of the RT complex into the cytoplasm [44]. CypA mediates HIV-1 attachment to target cells via heparans, and heparans in turn facilitate the interaction of CD147 and CypA [45]. Furthermore, the interaction of CD147 and CypA regulates HIV-1 infection in a signal-independent fashion [46].

Several lines of evidence indicate that CypA positively regulates the replication of HCV [47]. The PPIase activity of CypA assists the replication of HCV [48] and CypA increases the affinity of the polymerase to viral RNA via binding to NS5B, enhancing HCV replication [49]. In addition, CypA binds the HCV NS5A protein to aid viral replication [50]. Concerning HBV, CypA interacts with small surface proteins (SHBs) of the HBV surface antigen (HBsAg). In HBsAg-expressing cell lines, the expression level of CypA is lower than controls, and more CypA is secreted into the supernatant via the vesicular secretion pathway [19,51]. During VV infection, CypA stability is increased and CypA is translocated to the peripheral region of the nucleus, co-localizing with the sites of virus production. CypA is incorporated into the virus particle during morphogenesis and specifically localizes in the core [15]. In addition, CypA interacts with the nucleocapsid protein of VSV-NJ and VSV-IND in infected cells and is incorporated into the released virions of both serotypes. VSV-NJ utilizes CypA for post-entry intracellular primary transcription [14]. CypA is also reported to regulate SARS-CoV replication by binding to the nucleocapsid protein [52]. Recently, CypA was found to inhibit RV replication by facilitating host IFN-β production, which is independent of CypA PPIase activity, but dependent on the activation of the JNK signaling pathway [30].

## 4. CypA and Influenza Virus Infection

Host factors may play important roles in restricting cross-species transmission of influenza virus. Physical interaction methods have been developed to identify host factors that interact with viral components. These methods include yeast two-hybrid systems, proteomics analysis, cell-free reconstitution systems and a yeast-based influenza virus replicon system. To date, many host factors have been identified to interact with influenza viral proteins and take part in certain stages of the virus lifecycle. Influenza A virus M1 protein is the most abundant and relatively conserved protein in the viral particle, and core histones interact with the M1 protein [53]. M1 is phosphorylated by extracellular signal-regulated kinase (ERK) downstream of the Ras-activated factor (Raf)/mitogen-activated protein kinase (MEK)/ERK pathway. When cells are treated with a MEK-specific inhibitor after virus infection, NP and vRNP complexes accumulate in the nucleus [54]. The cellular receptor of activated C kinase (RACK) 1 may also interact with M1 and be involved in its phosphorylation [55]. Heat shock cognate (Hsc) 70 interacts with M1 protein, causing vRNP complexes to accumulate in the nucleus during heat shock [56]. Intracellular caspase-8 binds M1, which involves M1 in a caspase-8-mediated apoptosis pathway in influenza virus-infected cells [57]. Most importantly, we found that CypA interacts with M1 and has multiple functions in the lifecycle of inﬂuenza virus.

**Figure 1:**
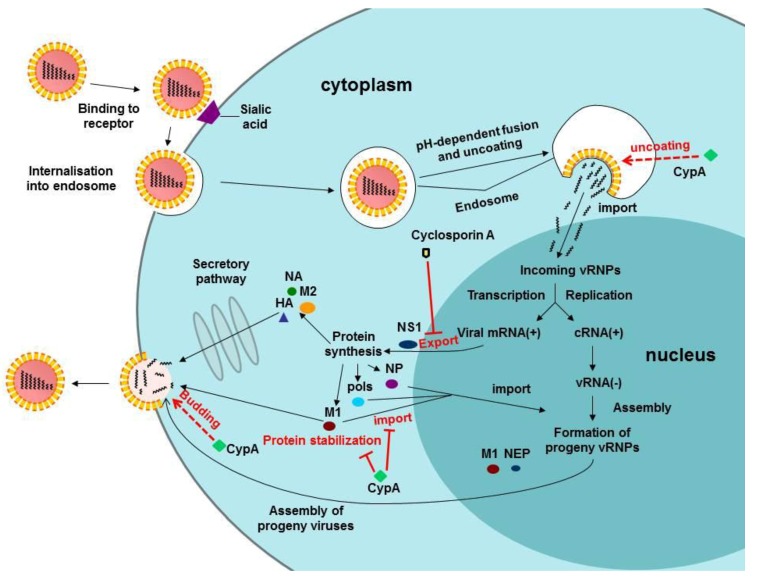
The functions of CypA in the lifecycle of influenza virus.

In 2008, CypA was shown to be in the core of the inﬂuenza virion [32] and is upregulated upon infection by avian H9N2 inﬂuenza virus in a human gastric carcinoma cell line (AGS) [58]. In 2009, we found that CypA is able to directly interact with the M1 protein of influenza virus, hindering its entry into the nucleus in the early stage of influenza virus infection (Figure 1), and avian CypA can also negatively regulate influenza virus replication [10]. We had confirmed that both CypA and CypA-R55A bound to the M1 protein, and there was no significant difference between CypA-transfected cells and CypA-R55A transfected cells in the viral titer levels. These results suggested that the isomerase activity of CypA is not necessary for viral replication in the cell level [9]. The effect of CypA on influenza virus replication was different from HIV and HCV, since the PPIase activity of CypA is required for viral replication. So, the precise functions and roles of CypA in the influenza virus lifecycle need further study. A cell line depleted of endogenous CypA was constructed to understand the precise functions of CypA in the influenza virus lifecycle. A one-step growth curve assay indicates that the first lifecycle of influenza virus in CypA knock-out cells is 2 h shorter than in control cells. Further studies indicate that CypA regulates influenza virus replication at the post-transcriptional level. However, CypA does not impair the nuclear export of viral mRNA. Indeed, CypA accelerates the degradation of the M1 protein by the ubiquitin proteasome system (UPS) (Figure 1) [11]. CypA degrades M1 via the proteasome-dependent pathway and, thereby, inhibits the replication of influenza virus at the post-translational level. These results suggest the struggle between virus offense and host defense via the UPS. Further studies are needed to identify the preferential ubiquitination site on M1 so that differences among the various M1 protein subtypes can be compared, which could also indicate the possible relationship between the different levels of infectivity in the numerous subtypes of influenza virus. CypA is firstly reported to play an important role in viral replication through accelerating the degradation of a viral protein [11]. Recently, the peptidyl-prolyl *cis/trans* isomerase Pin1, another member of the cyclophilin family, was reported to stabilize the human T-cell leukemia virus type 1 (HTLV-1) Tax oncoprotein and promote malignant transformation [59]. This suggests that the cyclophilin family may be involved in the regulation of viral replication at different stages by affecting the stability of various viral proteins. Thus, it is of interest to further study the detailed effect of the cyclophilins, with a focus on the proteasome system upon viral replication.

The well-known immunosuppressive drug CsA inhibits influenza virus replication through CypA-dependent and -independent pathways. In detail, CsA inhibits influenza virus replication at a post-transcriptional level and impairs the nuclear export of viral mRNA in the absence of CypA (Figure 1). In addition, the effect of CsA on influenza virus replication is independent of calcineurin signaling [12], because CsA partially inhibits influenza virus replication by regulating functional CypA. In addition, the immunosuppressive activity of CsA is not needed for its anti-influenza virus activity. Thus, we can develop CsA derivatives that have low toxicity and high activity as anti-influenza virus drugs.

As was reported, CypA is also incorporated into influenza virions [9,32]. However, the function of virion-associated CypA is still unclear. Recently, we found that the presence of CypA in the virions aids the infectivity of influenza virus particles, and CypA affects the ratio of M1/NP in the influenza virions (unpublished data). These results suggest that CypA may take part in the assembly of influenza virus and the uncoating process of the virus particles (Figure 1). However, how CypA is incorporated into the virions and how CypA assists in the infection of the virion requires further studies.

### Conclusion and Future Directions

Upon influenza virus infection, CypA acts at multiple steps of the viral lifecycle, including uncoating, viral protein entry into the nucleus, viral protein stability and assembly (Figure 1). In some stages, CypA negatively regulates influenza virus replication, but in other stages, CypA positively regulates the viral infection. Indeed, CypA is a cell-intrinsic regulator for influenza virus at different steps of its lifecycle.

Viral infection usually triggers host antiviral defenses, such as the interferon-I (IFN-I)-mediated antiviral response. Recently, CypA was found to play important roles in host antiviral innate immunity. For example, during HIV-1 infection, the interaction of CypA and newly synthesized HIV-1 CA assists HIV-1 to induce DC maturation, an antiviral type I interferon response, and the activation of T-cells. In addition, CypA inhibits Rotavirus replication by facilitating host IFN-β production. Whether CypA mediates host antiviral defenses during influenza virus infection is an interesting question and requires further study. In addition, CypA is secreted into the extracellular space, and the function of extracellular CypA during influenza virus replication deserves further study.
